# Diseases of the Heart and Aorta

**Published:** 1899-09

**Authors:** 


**Diseases of the Heart and Aorta.** By George Alexander Gibson, M.D. Pp. xx., 932. Edinburgh: Young J. Pentland. 1898.

A mastery of all that it seems capable of knowing in connection with diseases of the heart is displayed in this admirable treatise. A reference to standard works for information on some particular point as often as not fails to give satisfactory aid. In this book, however, we consider that not only knowledge, but exceptional judgment has been displayed. The mass of facts the book contains has been most ably handled, and rarely have we been disappointed in search for fresh light on a subject, or felt disposed to criticise where there is a summary of varied opinions.

One will always find points of minor importance with which one may not fully agree, such as the statement that when myocarditis is associated with pericarditis, "only the superficial layers of the heart" are affected. On two occasions during the past year we have seen myocarditis associated with rheumatic pericarditis extending through the wall of the left ventricle to the endocardial surface, where in one instance the speckled appearance produced by the degenerative changes was very marked. We refer to this point, because the subject of the power of rheumatism to attack the heart-muscle has recently been brought into prominence. We do not consider myocarditis associated with pericarditis to be due to direct spread of the inflammation from the pericardium inwards.

The varied aspects of disease are so numerous, that it must be rare to find descriptions that fully satisfy all observers. Dr. Gibson's book, however, seems to us to be as infallible as it is possible for a work of the kind to be.

